# The Influence of Motivation, Attitudes and Obstacles for Middle School Students’ Participation in Leisure Activities on Their Leisure Satisfaction in Southwest China

**DOI:** 10.3389/fpsyg.2021.758858

**Published:** 2021-12-09

**Authors:** Yunlan Wu, Jianan Sun, Falu Fan, Xia Wang, Yuanqiu Peng

**Affiliations:** ^1^School of Leisure Sport, Chengdu Sport University, Chengdu, China; ^2^School of Physical Education, University for Science and Technology Sichuan, Chengdu, China; ^3^The Department of Sport and Outward bound, SiChuan Technology and Business College, Chengdu, China; ^4^Chengdu Shuangshui Primary School, Chengdu, China; ^5^School of Physical Education, Southwest Medical University, Luzhou, China

**Keywords:** leisure motive, leisure barrier, leisure attitude, leisure satisfaction, structural equation mod

## Abstract

**Purpose:** To explore the relationship among leisure motivation, barriers, attitude and satisfaction of middle school students in Chengdu, Sichuan, to help students establish a positive leisure attitude and provide a reference for youth leisure counseling.

**Methods:** Based on consulting research literature, this paper designs a survey volume of teenagers’ leisure motivation, barriers, attitude, and satisfaction; 2249 valid questionnaires of middle school students in Chengdu were obtained by stratified random sampling. The data were statistically processed by the combination of exploratory factor analysis (EFA) and confirmatory factor analysis (CFA).

**Results:** (1) There are significant positive correlation effects between leisure motivation and leisure attitude, leisure attitude and leisure satisfaction, and leisure motivation and leisure satisfaction; (2) There is a low degree of positive correlation effect (*r* = 0.35 *) between leisure barriers and leisure motivation, which is contrary to common sense and needs to be further studied in the follow-up; (3) Leisure barriers has no significant direct impact on leisure satisfaction, but it can have a significant negative impact on leisure satisfaction with the intermediary variable of leisure attitude; (4) Leisure motivation is the most important variable in the whole leisure model structure. It not only has the greatest direct impact on leisure satisfaction but also has a great positive impact on leisure satisfaction through the intermediary of leisure attitude.

**Conclusion:** Adolescent leisure motivation, barriers, attitude, and satisfaction are complementary and interdependent. Among them, leisure motivation is the core variable and leisure attitude is the dual intermediary variable. Through the initiation of leisure motivation, helping adolescents establish a positive leisure attitude may be the key to ensure their leisure satisfaction.

## Introduction

Leisure life is the product of national economic growth and the change of social industrial structure. Due to the development of science and technology and socio-economic progress, the form of social life has changed significantly, which indirectly allows Chinese people to have more free time, and the demand and willingness for leisure activities have also increased. The rise of leisure activities has gradually become the focus of Chinese life, If leisure time is used to plan and improve leisure activities, it can not only bring personal health and relaxation benefits but also promote the beneficial interaction between people. Teenagers are the most energetic population in society. It is not only a critical period of personality development and life adaptation but also a stage with the greatest impact of physiological and psychological changes. The growth experience and the establishment of many ideas and behaviors in this period often have a decisive impact on their future personality development and behavior characteristics. At present, youth education is facing new opportunities and challenges. As an important educational activity, sports, leisure, and entertainment are inextricably linked with the educational theory itself. Research Report from sports, leisure, and entertainment educators: 11–16 years old is the most important stage to complete the socialization process of teenagers. If collective leisure and entertainment activities can be arranged at this stage, it is very important to train teenagers’ cooperation ability and team spirit. Leisure projects such as orienteering, multi-person rowing, and sailing are the best choice for this age group (Weybright et al., 2016; Song, 2017; Florian and Jörg, 2018).

Attitude plays an important role in forming personal behavior. A correct sports attitude can improve sports behavior, and attitude and behavior affect each other. Learning theory emphasizes that past behavior experience is also one of the factors forming attitude. Many scholars believe that leisure attitude is an individual’s response tendency for leisure, which represents an individual’s likes and dislikes for leisure activities and an individual’s readiness for leisure activities, and divides the structure of leisure attitude into three dimensions (Bailey et al., 2016; Holahan et al., 2017; Freire and Teixeira, 2018): (1) cognitive: including personal and social levels, refers to the knowledge and belief in leisure; Understand the belief of leisure and health, happiness and work relationship; The belief that leisure is beneficial to individuals’ relaxation and self-development, such as personal sexuality, expertise, the source of leisure information, etc.; (2) Affective: refers to an individual’s feeling of leisure, the degree of liking and disliking of leisure activities and experience, including the evaluation of leisure experience and activities, the degree of liking and dislike and direct and immediate feelings, such as personal values, expectations and interests; (3) Behavior: refers to the behavioral tendency of individuals to participate in leisure in the past, present and future, including the tendency to select leisure activities and choices; Past and present leisure activity participation status and experience.

Motivation is a force that urges people to take a certain behavior to meet a certain demand, because Motivation is the psychological or internal force that urges a person to carry out activities and it is the internal process that causes an individual activity and maintains the activity toward a certain goal (Weissinger and Bangalos, 1995). It is an internal driving force of an individual. Laroche et al. (2019) Based on previous research literature and theories (Downs et al., 2013; Ramey et al., 2016), proposed that leisure motivation is the psychological and social reasons for people to participate in leisure behavior, and divided leisure motivation into four-factor dimensions: (1) Intelligence: refers to the individual’s motivation to participate in leisure activities, including psychological activities of learning, exploration, discovery, creation or imagination; (2) Social: it means that the individual’s motivation to participate in leisure activities is for social reasons. It includes two basic needs: the needs of friendship and interpersonal relationship, and the needs of others’ respect; (3) competence-mastery: refers to the individual’s competence proficiency reason for participating in leisure activities, which is to achieve achievements, master, challenge, compete and master the characteristics of skilled activities, usually out of the instinct of the body; (4) Stimulus avoidance: the constituent elements are the motivation to escape and the living environment away from too much stimulation, and the need to pursue solitude and peaceful environment, as well as to rest and relax.

Leisure barriers are the hindrance of personal perception or experience, which is not necessarily the result of not participating in leisure activities, but may affect personal leisure preferences and change leisure participation. It can be seen that leisure barriers are related to an individual’s ability to overcome and deal with obstacles to successfully engage in leisure and has an impact on leisure experience and behavior (Jackson and Rucks, 1995). Cho and Price (2018) defined leisure barriers as individual subjective perception or reasons that affect individuals’ dislike or involvement in certain leisure activities, and summarized the influencing factors into three categories: (1) Individual barriers: refers to the psychological factors and states within an individual that affect his leisure preferences or participation, such as stress, anxiety, belief, etc.; (2) Interpersonal barriers: refers to the factors that affect an individual’s leisure preferences or participation due to lack of appropriate or sufficient leisure partners; (3) Structural barriers: refers to external factors that affect individual leisure preferences, such as resources, money, equipment, etc.

Leisure satisfaction is a subjective feeling that individuals affect their leisure experience. It is the concrete realization of motivation, preference, demand, or expectation (Rusbult et al., 1998). Rosa et al. (2019) pointed out that leisure satisfaction refers to the positive and good feelings obtained by individuals when participating in leisure activities, and the satisfaction of individuals with leisure experience and situation, and classified leisure satisfaction into six categories: (1) Psychological: Based on intrinsic motivation, individuals participate in freely selected activities and self-realization needs, so that individuals can show their individuality from leisure activities and seek self-expression; (2) Education: individuals pursue intellectual stimulation in participating in leisure activities, need new experiences to meet the curiosity of participants, and expand their personal life experience by learning new things and increasing knowledge; (3) Social aspect: individuals volunteer to participate in service groups or organizations to maintain the free choice of social relations. At the same time, the naturally formed interpersonal network is conducive to individual social interaction and communication, and obtaining social respect and respect from others; (4) Relaxation: Games and sports can restore vitality. Individual participation in leisure activities can enable individuals to have a full rest, relax and relieve the pressure and tension from work and life; (5) Physiologic: when individuals participate in leisure activities, some are physiologically challenging or maintain health, strengthen muscle and cardiopulmonary function, control weight and maintain good posture; (6) Aesthetic: the places where individuals participate in leisure activities are more satisfactory if the environment is beautiful, and make the leisure experience more interesting and pleasant.

The relationship between leisure satisfaction and leisure attitude, leisure motivation, leisure barriers, and other variables has long attracted the attention of scholars at home and abroad. Choi et al. (2017) found that leisure motivation and leisure attitude have a direct impact on leisure satisfaction respectively, and pointed out that there is a typical correlation between leisure attitude and leisure motivation; Soos et al. (2019) found that leisure attitude and leisure motivation are important factors affecting teenagers’ leisure behavior, and leisure attitude is its internal psychological factor, which is exposed externally through the stimulation of leisure motivation. Sukys et al. (2019) investigated college students and found that the correlation between leisure attitude and leisure barriers reached a significant level and was negatively correlated, while it was significantly positively correlated with leisure satisfaction. The higher the leisure identity, the higher the satisfaction from leisure experience. Hainey et al. (2013) found that leisure attitude and leisure motivation are important factors affecting teenagers’ leisure behavior. Leisure attitude is its internal psychological factor, which is exposed through the stimulation of leisure motivation. Zheng (2008) found that there was a significant negative correlation between leisure attitude and leisure barriers; Pu and Xu (2015) found that there is a significant positive correlation between leisure attitude and leisure satisfaction. Those who hold a higher leisure attitude can get higher leisure satisfaction from leisure experience; Wen (2019) found that leisure attitude has a direct and positive impact on leisure satisfaction, and believes that leisure satisfaction is the psychological satisfaction of leisure experience obtained by individuals engaged in leisure activities based on their attitude toward leisure. Chen (2019) found that leisure barriers are negatively correlated with leisure satisfaction. When leisure barriers increase, the satisfaction obtained from leisure decreases. Zhang et al. (2012) research proposed that the intensity of an individual’s motivation to engage in leisure is easily affected by barriers. If he thinks that engaging in leisure may be disturbed, his leisure intention and behavior will also be affected.

**FIGURE 1:**
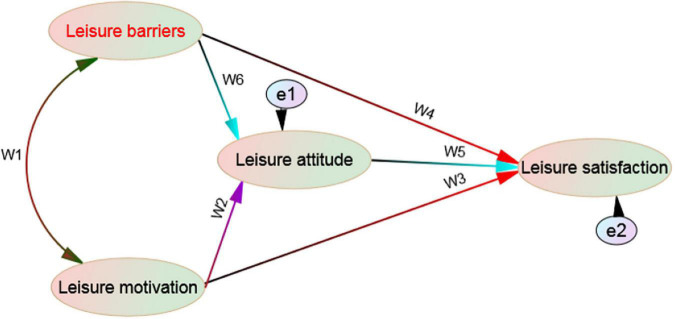
Hypothetical diagram of the relationship among leisure motivation, barriers, attitude and satisfaction.

To sum up, it is not difficult to find that attitude plays an important role in the formation of personal behavior. It is very necessary to enable middle school students to adjust their study and life through leisure activities and improve their cognition of leisure activities. By establishing a correct leisure attitude, we can achieve substantial results in the formation of leisure experience; Leisure motivation is the internal driving force to promote and maintain people’s activities. Understanding the reasons and motivation of individuals engaged in leisure activities can obtain the psychological motivation and tendency of individuals engaged in leisure activities. The stronger the leisure motivation, the higher the frequency of leisure participation; Leisure barriers is a factor affecting individuals to engage in leisure activities. The frequency of leisure participation and leisure barriers are negatively related, and leisure satisfaction is a positive psychological result after engaging in leisure activities, providing fascinating and unforgettable leisure experience. At present, although many scholars at home and abroad have discussed teenagers’ leisure attitude, leisure motivation, and leisure barriers, few scholars have a comprehensive and in depth understanding of the relationship between leisure satisfaction and leisure attitude, leisure motivation and leisure barriers. Understanding the relevant factors and relationships affecting teenagers’ leisure behavior, properly planning their leisure life, and appropriately engaging in leisure activities will have a positive impact on Teenagers’ school life and personality growth. Based on this, this study puts forward the following four hypotheses based on previous studies: (1) there are multiple groups of typical correlation structures among leisure motivation, leisure barriers, leisure attitude, and leisure satisfaction; (2) Leisure motivation has a positive impact on leisure attitude and leisure satisfaction, while leisure attitude also has a positive impact on leisure satisfaction; (3) The influence of leisure barriers on leisure attitude is negative, and the influence of leisure motivation on leisure attitude should be higher than that of leisure motivation on leisure satisfaction; (4) Leisure attitude plays an intermediary role in leisure barriers and leisure satisfaction. At the same time, leisure attitude also plays an intermediary mechanism between leisure motivation and leisure satisfaction (see Figure 1).

## Subjects and Research Design

First, junior high schools and senior high schools in Chengdu are stratified according to different districts, and then 5 schools are selected by random sampling. Each school is divided into grade 1 and grade 2 of junior middle school and grade 1 and grade 2 of senior high school (Note: considering the graduation class, grade three of junior middle school and senior high school are not the objects of the questionnaire). Students at each level are randomly selected for the questionnaire survey. A total of 2550 questionnaires are sent out, 2351 are recovered, and 102 invalid questionnaires are excluded, with an effective recovery rate of 88%. See Table 1 for sample distribution.

**TABLE 1:** Statistical table of sample distribution in different regions.

Academic name	Questionnaire (*N* = 2351)			Valid sample (*N* = 2249)		Junior middle school		Senior high school	
	Distribute	Regain	Delete	Male	Female	Grade 1	Grade 2	Grade 1	Grade 2
Sichuan normal affiliated middle school	510	469	16	51.3%	48.7%	26.5%	25.6%	22.7%	25.2%
Chengdu No.17 middle school	510	475	31	49.6%	50.4%	24.8%	25.9%	26.1%	23.2%
Shishi United middle school	510	457	25	50.9%	49.1%	25.6%	26.7%	25.9%	21.8%
Chengdu Lianxin middle school	510	471	30	53.7%	46.3%	27.1%	25.6%	24.9%	22.4%
Chengdu Yandaojie middle school	510	479	0	52.5%	47.5%	25.9%	27.1%	25.4%	21.6%
Total	2550	2351	102	1160	1089	581	589	563	516

### Research Methods

#### Questionnaire Survey Method

The whole questionnaire consists of basic data of subjects and four scales: The basic data of the subjects include gender, age, grade of study, accommodation, monthly discretionary funds for leisure activities, average academic achievement, and family residence. Among the four scales, The measurement of leisure attitude is compiled by Wei and Liu (2013), which is divided into three dimensions with a total of 16 items, namely leisure cognition (e.g., leisure activities are beneficial to personal health), leisure behavior (e.g., I often engage in leisure activities) and leisure emotion (e.g., my leisure activities are novel and interesting); The leisure motivation scale is compiled by Wang (2007), which contains 4 dimensions and 21 items in total, namely, develop intelligence (e.g., I participate in leisure to stimulate imagination or cultivate creativity), social skills (e.g., I participate in leisure to establish and maintain good friendship), competent and skilled (e.g., I want to gain a sense of achievement by participating in leisure activities) And stimulus escape (e.g., I participate in leisure activities to escape crowded and noisy daily life); The leisure barriers scale was compiled by Zhao (2003), which contains three dimensions and 17 items in total, namely internal obstacles (for example, I am too shy and introverted to engage in leisure activities), internal barriers (for example, people I know usually don’t have good skills, so they can’t engage in leisure activities with me), and junction observation (for example, if I have transportation, I am more likely to engage in leisure activities); The leisure satisfaction scale was compiled by Chen (2008), which contains 6 dimensions and 25 items in total, namely mental health (for example, participating in leisure activities makes me very interested), education happy (for example, participating in leisure activities can increase my knowledge), social satisfaction (for example, participating in leisure activities can give me opportunities to interact socially with others), pressure relief (for example, participating in leisure activities can help me relax my body and mind) Physical health (for example, participating in leisure activities can help me recover my strength) and field aesthetic feeling (for example, participating in leisure activities can let me enjoy the beauty of nature). The five-point Likert-scaled items are adopted for the above scales, which are divided into very disagree, disagree, uncertain, agree, and very agree, with 1 to 5 points respectively.

#### Questionnaire Validity and Reliability

On March 15, 2019, this study selected two classes in Chengdu Shishi middle school and Jinniu middle school respectively, distributed 300 questionnaires, recovered 278 questionnaires, deducted 12 invalid questionnaires and 266 valid questionnaires. The validity and reliability of the questionnaire were tested and corrected based on the pre-test. The formal investigation was completed from September 15 to 30, 2019.

Table 2 shows:

**TABLE 2:** Validity and reliability test of attitude, motivation, hindrance, and satisfaction scale of leisure activities.

Scale name	KMO and bartlett ball test	Number of items included	Dimension name	Composite reliability	Cronbach α coefficient
Leisure attitude scale	KMO = 0.87	6	Leisure cognition	0.80	0.84
	*P* = 0.000	5	leisure behavior	0.85	0.81
		5	leisure emotion	0.81	0.83
Verification results of scale 1: AGFI = 0.92, CFI = 0.95, NFI = 0.93, IFI = 0.91, RMSEA = 0.03; The overall cronbach’s α coefficient is 0.85.					
	KMO = 0.78	6	Develop intelligence	0.82	0.79
Leisure Motivation Scale	*P* = 0.000	5	Social skills	0.81	0.78
		5	Competent and skilled	0.87	0.85
		5	Stimulus escape	0.84	0.80
Verification results of scale 2: AGFI = 0.91, CFI = 0.92, NFI = 0.92, IFI = 0.93, RMSEA = 0.04; The overall cronbach’s α coefficient is 0.88.					
	KMO = 0.85	6	Internal Obstacles	0.88	0.86
Leisure barriers scale	*P* = 0.000	5	Interpersonal barriers	0.80	0.82
		6	Junction obstruction	0.79	0.83
Verification results of scale 3: AGFI = 0.94, CFI = 0.90, NFI = 0.91, IFI = 0.93, RMSEA = 0.02; Overall Cronbach’s α coefficient 0.85.					
	KMO = 0.82	5	mental health	0.86	0.84
Leisure satisfaction scale	*P* = 0.000	4	Education happy	0.83	0.81
		4	Social satisfaction	0.81	0.89
		4	Pressure relief	0.87	0.86
		4	Physical health	0.82	0.85
		4	Field Aesthetic feeling	0.86	0.81
Verification results of scale 4: AGFI = 0.91, CFI = 0.93, NFI = 0.95, IFI = 0.92, RMSEA = 0.03; The overall cronbach’s α coefficient is 0.84.					

Exploratory factor analysis (EFA) showed that the sports leisure attitude scale with 16 items was suitable for factor analysis (KMO = 0.87, Bartlett’s test for sphericity *p* < 0.001), and three common factors could be extracted, and the corresponding Cronbach’ α coefficients were 0.84, 0.81, and 0.83 respectively. In addition, the overall scale α coefficient = 0.85; Confirmatory factor analysis (CFA) showed that the fitness indexes AGFI, CFI, NFI, and IFI were 0.92, 0.95, 0.93, and 0.91, which were all greater than the standard of 0.90, RMSEA = 0.03 (less than 0.05, with good adaptation); In addition, the combined reliability of the three common factors (potential variables) is more than 0.79, which shows that the scale has good reliability and validity.

Exploratory factor analysis showed that the leisure motivation scale with 21 items was suitable for factor analysis (KMO = 0.78, Bartlett’s test for sphericity *p* < 0.001). The scale could extract 4 common factors, corresponding to Cronbach’ α coefficient is between 0.79–0.85, and the overall scale α Coefficient = 0.88; Confirmatory factor analysis showed that the fitness indexes AGFI, CFI, NFI, and IFI were 0.91, 0.92, 0.92, and 0.93, which were all greater than the standard of 0.90, RMSEA = 0.04 (less than 0.05, good adaptation); In addition, the combined reliability of the four dimensions (potential variables) is more than 0.81, which shows that the reliability and validity of this scale are good.

Exploratory factor analysis showed that the leisure barriers scale with 17 items was suitable for factor analysis (KMO = 0.85, Bartlett ball test *p* < 0.001). The scale could extract three common factors, corresponding to Cronbach’ α coefficient is between 0.82–0.86, and the overall scale α Coefficient = 0.85; Confirmatory factor analysis showed that the fitness indexes AGFI, CFI, NFI, and IFI were 0.94, 0.90, 0.91, and 0.93, which were all greater than the standard of 0.90, RMSEA = 0.02 (less than 0.05, good adaptation); In addition, the combined reliability of the three dimensions (potential variables) is more than 0.79, which shows that the reliability and validity of this scale are good.

Exploratory factor analysis showed that the leisure satisfaction scale with 25 items was suitable for factor analysis (KMO = 0.82, Bartlett’s test for sphericity *p* < 0.001). The scale could extract 6 common factors, corresponding to Cronbach’ α coefficient is between 0.81–0.86, and the overall scale α Coefficient = 0.84; Confirmatory factor analysis showed that the fitness indexes AGFI, CFI, NFI, and IFI were 0.91, 0.93, 0.95, and 0.92 in order, which was all greater than the standard of 0.90, RMSEA = 0.03 (less than 0.05, good adaptation); In addition, the combined reliability of the six dimensions (potential variables) is more than 0.81, which shows that the reliability and validity of this scale are good.

### Mathematical Statistics

Canonical correlation analysis is a statistical method used to test the correlation degree between one group of control variables and another group of criterion variables. It aims to find the maximum correlation between the linear combination of control variables and the linear combination of criterion variables. Therefore, canonical correlation analysis tests the canonical correlation combination of multiple criterion variables and multiple control variables, Canonical correlation analysis can produce a combination of significant and insignificant canonical correlation. Generally, it can provide the following basic information: one is the typical correlation coefficient can reflect the correlation degree between the linear combination of control variables and the linear combination of standard variables. The typical correlation coefficient must reach a significant level to represent the significant correlation between the two groups of linear combinations. The second is the judgment coefficient (i.e., the square value of typical correlation coefficient *R*). It means that the typical factors of the standard variable can be explained by the typical factors of the control variable (not less than 10%). The third is the structural coefficient (typical load). It is intended to control the correlation between the variable and the criterion variable to their respective typical linear combinations. The absolute value of the coefficient must be more than 0.30 to explain that their respective typical linear combinations have explanatory power.

SPSS17.0 and Amos version 17.0 statistical analysis software were used to statistically process the survey data by using the methods of canonical correlation analysis, exploratory factor analysis (EFA) and confirmatory factor analysis (CFA). Explore and calculate the mediating effect of physical activity according to the bootstrap method (Liu et al., 2017). In this study, non-parametric percentile bootstrap was used to test the significance of mediating effect. The original data were sampled 2000 times and 95% confidence interval (CI) was estimated. Firstly, it is judged that the indirect effect does not contain 0 within the 95% confidence interval and reaches a significant level, indicating that there is an intermediary effect. At this time, if the direct effect contains 0 within the 95% confidence interval, it means that the direct effect is not significant and is a complete intermediary effect; If the indirect effect and direct effect do not include 0 in the 95% confidence interval, both reach a significant level, and the total effect does not include 0 in the 95% confidence interval, reaching a significant level, it is a partial intermediary effect. The significance level of all indicators was set as α = 0.05.

## Results

### Analysis of the Typical Relationship Among Teenagers’ Leisure Barriers, Attitudes, Motivation, and Satisfaction

**FIGURE 2:**
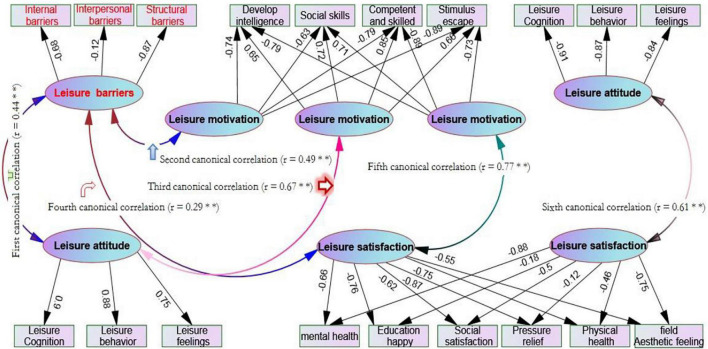
Typical correlation among leisure motivation, attitude, barriers, and satisfaction.

Figure 2 shows that there are six groups of typical correlations among leisure barriers, leisure attitude, leisure motivation, and leisure satisfaction:

The first canonical correlation reflects the relationship between leisure attitude (control variable) and leisure barriers (criterion variable). The canonical correlation coefficient *r* = 0.44^∗∗^ and reached a significant level, and the determination coefficient *R*^2^ = 0.194, indicating that the canonical factors in the control variable group can explain 19.4% of the total variation of canonical factors in the standard variable group (exceeding the minimum standard of 10%). In the control variable group, leisure cognition, leisure behavior, and leisure emotion were highly correlated with leisure attitude, and the typical factor loads were 0.90, 0.88, and 0.75 respectively. Therefore, it can be considered that leisure attitude affects leisure barriers through leisure cognition, behavior, and emotion in its variable group, while the variables highly related to leisure barriers are internal barriers and structural barriers, and the corresponding loads are −0.68 and −0.87 in turn; From the positive and negative signs of factor load, the relationship between the two is reversed.

The second group of canonical correlation reflects the relationship between leisure barriers (control variable) and leisure motivation (criterion variable). Its canonical correlation coefficient *r* = 0.49^∗∗^ and reaches a significant level, and its determination coefficient *R*^2^ = 0.24, indicating that the canonical factors in the control variable group can explain 24% of the total variation of canonical factors in the criterion variable (exceeding the minimum standard of 10%). In the control variable group, intrinsic and structural barriers have a high correlation with leisure barriers, and their typical factor loads are −0.68 and −0.87 respectively. Therefore, it can be considered that leisure barriers mainly affect leisure motivation by the intrinsic and structural barriers in the variable group, while the variables highly correlated with leisure motivation are the development of intelligence, social skills, proficiency, and stimulus avoidance, The corresponding loads are −0.74, −0.63, −0.79, and −0.89; From the positive and negative signs of factor load, the relationship between the two is in the same direction.

The third group of canonical correlations reflects the relationship between leisure motivation (control variable) and leisure attitude (criterion variable). The canonical correlation coefficient *r* = 0.67^∗∗^, reaching a significant level, and the determination coefficient *R*^2^ = 0.45, indicating that the canonical factors in the control variable group can explain 45% of the total variation of canonical factors in the standard variable (exceeding the minimum standard of 10%). Among the control variables, the development of intelligence, social skills, competence, proficiency, and stimulus avoidance is highly correlated with leisure motivation, and the typical factor loads are 0.65, 0.72, 0.85, and 0.66 respectively. Therefore, it can be considered that leisure motivation affects leisure attitude through the development of intelligence, social skills, competence, proficiency, and stimulus avoidance, while the variables highly correlated with leisure attitude are leisure cognition The corresponding loads of behavior and emotion were 0.90, 0.88, and 0.75 respectively; From the positive and negative signs of factor load, the relationship between the two is in the same direction.

The fourth group of canonical correlation reflects the relationship between leisure barriers (control variable) and leisure satisfaction (criterion variable). Its canonical correlation coefficient *r* is 0.29^∗∗^, reaching a significant level, but the determination coefficient *R*^2^ is only 0.08, indicating that the canonical factors in the control variable group can only explain 8% of the total variation of canonical factors in the criterion variable and fail to reach the minimum standard of 10%. Therefore, it can be considered that the correlation between leisure barriers and leisure satisfaction is weak, and the impact on each other is limited.

The fifth group of canonical correlation reflects the canonical correlation between leisure motivation (control variable) and leisure satisfaction (criterion variable). Its canonical correlation coefficient *r* = 0.77^∗∗^ and reaches a significant level, and the determination coefficient *R*^2^ = 0.59, indicating that the canonical factors in the control variable group can explain 59% of the total variation of the canonical factors of the criterion variable (exceeding the minimum standard of 10%). In the control variable group, the development of intelligence, social skills, competency proficiency, and stimulus avoidance is highly correlated with leisure motivation, and the typical factor loads are −0.79, −0.71, −0.89, and −0.73 respectively. Therefore, it can be considered that leisure motivation affects leisure satisfaction through the four dimensions of development intelligence, social skills, competency proficiency, and stimulus avoidance in the variable group, The variables highly correlated with leisure satisfaction were mental health, educational pleasure, social satisfaction, stress relief, physical health, and site aesthetics, and the corresponding loads were −0.66, −0.76, −0.62, −0.87, −0.75, and −0.55 respectively; From the positive and negative signs of factor load, the relationship between the two is in the same direction.

The sixth group of canonical correlation reflects the relationship between leisure attitude (control variable) and leisure satisfaction (criterion variable). The canonical correlation coefficient *r* = 0.61^∗∗^, reaching a very significant level, and the determination coefficient *R*^2^ = 0.37, indicating that the canonical factors in the control variable group can explain 37% of the total variation of canonical factors in the standard variable group (exceeding the minimum standard of 10%). In the control variable group, leisure cognition, behavior, and emotion are highly correlated with leisure attitude, and their typical factor loads are −0.91, −0.87, and −0.84, respectively. Therefore, it can be considered that leisure attitude affects leisure satisfaction through cognition, behavior, and emotion in the variable group, while the variables highly correlated with leisure satisfaction are mental health, social satisfaction, physical health, and site aesthetics, The corresponding loads are −0.88, −0.50, −0.46, and −0.75; From the positive and negative signs of factor load, the relationship between the two is in the same direction.

#### Structural Equation Model Analysis of Leisure Barriers, Motivation, Attitude and Satisfaction Structural Model Verification

Table 3 shows:

**TABLE 3:** Statistical table of fitness test for model evaluation (*P < 0.05, indicating that the model is not suitable; *P* > 0.05, indicating adaptation).

	Absolute fit test						Increment fit test			
	*X* ^2^	*X*^2^/df	*P*	RMSEA	GFI	AGFI	NFI	IFI	CFI	RFI
Initial model	81.01	9.17*	0.000	0.341	0.817	0.808	0.787	0.801	0.830	0.785
Modified model	5.88	1.89	0.081	0.054	0.925	0.930	0.932	0.941	0.934	0.945

From the results of absolute fit test: the initial mode absolute fit index *x*2 = 81.01, *X*2/DF = 9.17, *P* = 0.000 < 0.05, indicating that the covariance matrix of the hypothetical model is not well matched with the observed data (generally, the value of *x*2/DF should be between 1 and 3); GFI = 0.817 (> 0.90 is the adaptation), AGFI = 0.808 (> 0.90 is the adaptation), RMSEA = 0.341 (generally, RMSEA < 0.05 is excellent, and 0.05 ∼ 0.08 is good). From the value-added adaptation test results, NFI = 0.787 (adaptation > 0.90), IFI = 0.801 (adaptation > 0.90), CFI = 0.830 (adaptation > 0.90), RFI = 0.785 (adaptation > 0.90). In short, whether absolute fit or Increment fit test, the initial correlation model of this study is not well matched with the actual data, so the correlation model must be corrected.

**FIGURE 3:**
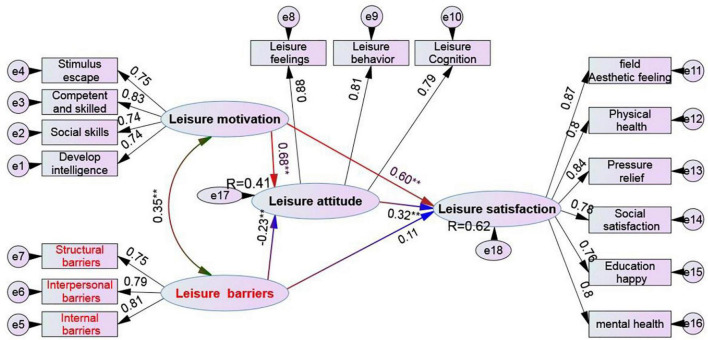
Overall structural equation model of leisure barriers, motivation, attitude, and satisfaction.

According to the path suggested by the correction index in the initial model, this study modifies the initial model with the original theoretical framework and adds the covariance relationship between the measurement index error terms (e2-e5, e1-e8, e2-e4, e11-e6, e1-e6, e2-e9) one by one. The results show that the absolute adaptation index of the modified model is *X*^2^ = 5.88, *X*^2^/DF = 1.89, *P* = 0.081 > 0.05, indicating that the covariance matrix of the model is adapted to the observed data (*X*^2^/DF = 1.78 is adapted between 1 and 3); GFI = 0.925 (> 0.90 for adaptation), AGFI = 0.930 (> 0.90 for adaptation), RMSEA = 0.054 (good at 0.05∼0.08). From the increment fit test results, NFI = 0.932 (adaptation > 0.90), IFI = 0.941 (adaptation > 0.90), CFI = 0.934 (adaptation > 0.90), RFI = 0.945 (adaptation > 0.90). It can be seen that the initial correlation model of this study is well adapted to the actual data after correction (see Figure 3).

### Analysis of the Dual Mediating Role of Leisure Attitude

Figure 3 shows in combination with Table 4:

**TABLE 4:** Analysis of leisure attitude as a dual intermediary effect.

Intermediary model I: leisure motivation → leisure attitude → leisure satisfaction			Intermediary model II: leisure barriers → leisure attitude → leisure satisfaction		
	Standardization coefficient	95% confidence interval		Standardization coefficient	95% confidence interval
Indirect effect: leisure motivation → leisure attitude → leisure satisfaction	0.22**	0.102∼0.411	Indirect effect: leisure barriers → leisure attitude → leisure satisfaction	−0.07**	−0.158∼−0.008
Direct effect: leisure motivation → leisure satisfaction	0.60**	0.326∼0.781	Direct effect: leisure barriers → leisure satisfaction	0.01	−0.109∼0.159
Total effect: leisure motivation → leisure satisfaction	0.82**	0.501∼0.902	Total effect: leisure barriers → leisure satisfaction	−0.06**	−0.216∼−0.008

Attachment: direct effect, indirect effect and total effect are directly derived from Amos. *, **, and *** represents significant levels of 0.05, 0.01 and 0.001 respectively.

The indirect effect of leisure motivation on middle school students’ leisure satisfaction is 0.22^∗∗^, which is very significant. The confidence interval of 0.102∼0.411 does not contain zero, while the direct effect of leisure motivation on leisure satisfaction = 0.60^∗∗^, which is very significant. The confidence interval of 0.326∼0.781 does not contain zero. At the same time, the total effect of leisure motivation on leisure satisfaction = 0.82^∗∗^ and the confidence interval of 0.501∼0.902 also does not contain zero, This fully affirms that leisure attitude plays a partial intermediary role between leisure motivation and leisure satisfaction. The other path is the impact of leisure barriers on leisure satisfaction, in which the indirect effect of leisure barriers on leisure satisfaction is −0.07^∗∗^, reaching a significant level, with a confidence interval of −0.158 ∼−0.008, obviously excluding zero, while the direct effect of leisure barriers on middle school students’ leisure satisfaction = 0.01, not significant, with a confidence interval of −0.109∼0.159, obviously including zero. Therefore, we can judge that leisure attitude plays a complete intermediary role between leisure barriers and leisure satisfaction.

It can be further found from Figure 3 that the value of the judgment coefficient of the impact of leisure motivation and leisure barriers on middle school students’ leisure attitude is *R*^2^ = 0.41, which shows that 41% of the variation of middle school students’ leisure attitude can be explained by leisure motivation and leisure barriers. Among them, the impact of leisure motivation on leisure attitude is positive, and the explanation strength is about 46% (from the standardized path coefficient *r* = 0.68^∗∗^, 0.68 × 0.68 = 0.46), while the impact of leisure barriers on leisure attitude is negative, and the explanation is about 5% (from the standardized path coefficient *r* = −0.23^∗∗^, 0.23 × 0.23 = 0.05), the influence of leisure motivation on leisure attitude is significantly higher than that of leisure barriers. In the influence of leisure motivation, leisure barriers, and leisure attitude on middle school students’ leisure satisfaction, the judgment coefficient *R*^2^ = 0.62, which shows that 62% of the variation of middle school students’ leisure satisfaction can be explained by the direct or indirect influence of leisure motivation, leisure barriers, and leisure attitude. The direct influence of leisure motivation, leisure barriers and leisure attitude on leisure satisfaction was 36% (0.60 × 0.60 = 0.36), 10% (0.32 × 0.32 = 0.10), 1% (0.11 × 0.11 = 0.01) respectively, intermediary I influence 22% (0.68 × 0.32 = 0.22), the influence of complete mediation II is negative, and the size is 7% (−0.23 × 0.32 = −0.07). The overall influence is positive (direct influence + indirect influence), and the size is 62%.

## Discussion

In the first group of canonical correlations, the absolute values of the structural coefficients of the control variables’ leisure attitude are more than 0.70, indicating that each control variable has a high degree of correlation with leisure attitude; In the criterion variable leisure barriers, except interpersonal barriers, the absolute values of the structural coefficients of personal internal barriers and structural barriers are greater than 0.6. It can be seen that leisure cognition, behavior, and emotion are the main factors affecting personal internal barriers and structural barriers. That is, the subjects’ cognition, feeling, and preference for leisure activities and experience, as well as all their leisure behavior patterns, It will affect their personality traits and mental state, leisure preferences, and leisure participation. In addition, from the perspective of leisure attitude, the structural coefficient symbols of each control variable and each leisure barriers criterion variable are reversed, indicating that the better the subjects’ leisure attitude, the lower the barriers they encounter in leisure. This conclusion is the same as Kyunghyun et al. (2017) and Tan (2017). Figure 2 of the structural model clearly shows that leisure barriers have a certain negative impact on leisure attitudes, and the standardized path coefficient is −0.23^∗∗^, which also supports the research results of Sylvester et al. (2018). However, the results of this study do not support the findings of Hofer et al. (2011) that leisure barriers may not completely limit individuals’ leisure attitude (behavior) but do affect individuals’ choices and experience in participation. If individuals have high intrinsic motivation for leisure, they can try to overcome difficulties and achieve leisure needs even in the face of leisure barriers. Therefore, this result needs further research in the future. 28–29.

The second group of canonical correlation showed that among the control variables, individual internal barriers and structural barriers were closely related to typical factors leisure barriers, among which structural barriers had the best correlation (*r* = −0.87); In terms of the criterion variable leisure motivation, the absolute value of the structure coefficient of each dimension is greater than 0.6, and the correlation between stimulus avoidance and leisure motivation is the highest (*r* = −0.89). This typical relationship reveals that personal internal obstacles and structural obstacles significantly affect leisure motives such as developing intelligence, social skills, competence, proficiency, and stimulus avoidance. Since the structural coefficients of each dimension of leisure barriers and leisure motivation are negative, the correlation coefficients of the two are shown in Figure 3 of the structural equation (r = 0.35^∗∗^), so it is certain that the lower the degree of leisure barriers suffered by teenagers, the lower their motivation to engage in leisure. This result also does not support the view of scholar Tan (2017). Perhaps leisure barriers does not only interfere with leisure behavior, but may have a positive interaction with the feeling of happiness and satisfaction brought by leisure. Arie and Tal (2013) pointed out that leisure barriers can not only promote and form leisure experience, but also become the driving force to stimulate leisure and promote the positive feeling of leisure. If individuals simply remove leisure barriers in the process of leisure, it will have a negative impact on the formation of leisure experience.

The third canonical correlation shows that the development of intelligence, social skills, competence, proficiency and stimulus avoidance in the variable group of leisure motivation are highly correlated with them (*r* exceeds 0.65), while the factor load of structural factors cognition, behavior and emotion of leisure attitude in the variable group exceeds 0.74, and the symbol of factor load of leisure motivation and leisure attitude is the same direction. Figure 3 of the structural model shows that leisure motivation has a very high direct impact on leisure attitude (β = 0.68^∗∗^). This fully affirms that the higher the individual’s leisure motivation, the stronger their leisure attitude. This result is consistent with the research findings of McDavid et al. (2014) and Seghers et al. (2014). In addition, Axel (2013) took teenagers as the research object and found that leisure attitude and leisure motivation are important factors affecting their leisure behavior. Leisure attitude is an internal psychological factor, which is revealed in appearance through the stimulation of leisure motivation; Namho et al. (2014) put forward the hierarchical model of internal and external motivation according to self-determination theory and relevant research, which also indicates that different motivation forms will affect individual cognition, emotion, and behavior. Therefore, the significant positive relationship between leisure motivation and leisure attitude should be confirmed.

The fourth canonical correlation means that the canonical correlation between leisure barriers and leisure satisfaction is not strong (*r* = 0.287^∗∗^) and the structural model Figure 3 also shows that the causal relationship is weak (β = 0.11), but it can harm leisure satisfaction through leisure attitude, which is consistent with the research results of Zhang and Yi (2013). However, previous studies on leisure barriers focused on the results of individuals’ non-participation in leisure activities caused by their cognition of leisure barriers. That is, leisure barriers is the reason why personal perception or experience is hindered or affected to engage in leisure activities. Leisure barriers does not necessarily lead to the result of not participating in leisure activities but may affect personal leisure preferences and change leisure participation. Therefore, leisure barriers is related to an individual’s ability to deal with barriers when successfully engaging in leisure and has an impact on leisure attitude (Zhang et al., 2017). It seems that in the process of leisure, facing the leisure barriers and paying for it, leisure satisfaction is not completely negative. It is necessary to conduct follow-up research to explore the linear relationship between the two variables.

The fifth group of canonical correlation shows that among the control variables of leisure motivation, the absolute values of the structural coefficients of developing intelligence, social skills, competence and skill, and stimulus avoidance are greater than 0.75, indicating that each dimension of the control variable is highly correlated with leisure motivation, and the absolute values of each dimension and its structural coefficient of leisure satisfaction are also greater than 0.65, so it is considered that intellectual development Leisure motivation structures such as social skills, competence and proficiency, and stimulus avoidance can significantly affect the six dimensions of leisure satisfaction. Figure 3 of the structural model further confirms the direct influence of leisure motivation on leisure satisfaction, and the standardized path coefficient is β = 0.60^∗∗^. That is, to improve intelligence, establish interpersonal relationships and gain respect from others, and challenge the limits to achieve various skills, individuals often need to escape or stay away from the overstimulated life state, and leisure sports are the most suitable. Through leisure participation, we can benefit from personal psychology, obtain good social interaction with others, help understand others and surrounding things, relieve excessive life and academic pressure, develop physical fitness and obtain the best health methods.

The sixth canonical correlation shows that in the leisure attitude variable group, leisure cognition, behavior, and emotion are highly correlated with leisure attitude (*r* more than 0.80), while leisure satisfaction is only highly correlated with mental health, aesthetic feeling, physiological and social satisfaction. The sign of the load of the two factors shows the same direction, which shows the subjects’ cognition of leisure activities and experience Feelings and preferences and all leisure behavior patterns will affect their leisure preferences and interest tendencies in open space and natural beauty, building interpersonal interaction and leisure activities. This is consistent with the research results of Badia et al. (2011) and Kono et al. (2020), that is, teenagers who hold a highly recognized leisure attitude toward leisure can get higher leisure satisfaction from their leisure experience. Figure 3 shows that the direct influence of leisure attitude on leisure satisfaction is low (β = 0.32^∗∗^). According to Hamm and Yun (2018), among the three dimensions of leisure cognition, behavior, and emotion, leisure behavior has the lowest score, and perceived benefits cannot effectively predict physical activity behavior. It is further pointed out that sports benefits do not necessarily lead to physical activity behavior. It can be seen that high attitude may only lead to high cognition and emotion, which may not lead to leisure activity “behavior”, That is, subjects cannot experience leisure fun without taking action, and satisfaction is naturally limited, which may be part of the reason why leisure attitude has a little direct effect on leisure satisfaction.

The overall structure model of leisure motivation, barriers, attitude, and satisfaction (Figure 3) shows that leisure motivation is the most important pre-variable. Through leisure attitude, leisure motivation has a significant impact on leisure satisfaction. Leisure attitude plays an important intermediary role. However, the joint explanation variation of leisure motivation and leisure barriers on leisure attitude is 41%, but the impact of leisure barriers on leisure attitude is negative. It can be seen that leisure motivation plays a dominant and dominant role in the impact of leisure attitude. For example, Mackenzie et al. (2018) pointed out that the internal motivation of leisure is the tendency of individuals to seek internal reward from leisure behavior, and it is the internal force that triggers individuals to engage in leisure activities. Zhang (2008) pointed out that leisure provides many benefits, which can adjust stress, provide positive mood, reduce negative emotion and physiological mechanisms. When individuals perceive the benefits of leisure, the internal motivation of leisure is born, because the benefits of leisure will promote individuals to participate in leisure activities, It leads to a positive relationship between leisure interests and leisure intrinsic motivation. In other words, if leisure participants can have a good experience in the process of activities, they can not only enhance their interest and love of leisure activities but also be more willing to continue to participate. When individuals have stronger leisure motivation, their leisure experience will be better, and leisure satisfaction will naturally increase with their personal experience. The results of this study echo these views.

From the perspective of the joint effect of the three variables of leisure attitude, leisure motivation and leisure barriers on leisure satisfaction, the joint explanation variation is 62%, but leisure motivation and leisure attitude are positively correlated with leisure satisfaction, while leisure barriers has a little direct impact on leisure satisfaction, while the direct impact of leisure attitude on leisure satisfaction is limited (about 10%), Therefore, it can be inferred that the main influence on leisure satisfaction is leisure motivation, which has a direct influence of 36%, and it also has an indirect influence of 22% through the intermediary of leisure attitude. As Ryan and Glendon (1998) said, leisure motivation emphasizes that individuals seek the most ideal reasons to engage in leisure to meet the needs of their internal value, such as relaxation or escape, to stimulate their motivation to participate in leisure activities. The essence of leisure satisfaction lies in the pursuit of personal choice, through the realization of personal internal leisure motivation and demand, to form the degree of satisfaction obtained by individuals engaged in leisure. Based on the important role of leisure motivation, this study suggests that at present, the decision-makers of all kinds of senior middle schools in China should pay more attention to students’ leisure education, establish a correct concept of leisure and assist in appropriate leisure planning. When students encounter barriers in leisure, they should assist students to face the barriers and strive to overcome them, to stimulate their motivation to engage in leisure, Enhance the intensity of their leisure motivation, to enhance the satisfaction of leisure.

## Problems and Prospects

In order to improve and increase middle school students’ leisure satisfaction, we should make good use of the factors affecting leisure activities. The results of this study infer that as long as it can actively stimulate middle school students’ leisure motivation and help them establish a positive leisure attitude, it should improve students’ leisure satisfaction and improve their quality of life. Therefore, it is necessary to strengthen middle school students’ leisure education, establish correct leisure concepts and attitudes, emphasize leisure benefits and induce their leisure motivation.

In terms of leisure motivation, schools should add leisure facilities, beautify the leisure environment and strengthen the safety of leisure venues, which will be of positive help to students’ participation in after-school leisure; In terms of leisure barriers, we should provide leisure consulting and leisure counseling services, increase students’ leisure sports knowledge and skills through appropriate leisure sports courses and leisure sports related activities, and regularly handle leisure sports lectures, so as to reduce the increase of students’ leisure barriers, which will create a virtuous circle for the overall leisure model of teenagers (Madariag and Romero, 2016; Villar et al., 2017).

There are many factors and levels involved in the causal model affecting middle school students’ leisure. This study only discusses the causal model from the variables such as leisure attitude, leisure motivation, leisure barriers, and leisure satisfaction. However, in order to fully understand the overall picture of complex leisure model, more influencing variables should be added appropriately, When verifying similar hypothetical models in the future, we should consider adding different variables to the impact model in order to obtain more complete leisure model information.

The survey participants of this study are mainly middle school students in Chengdu in the western region. Therefore, the follow-up researchers should cover the subjects in the northern, central, southern and eastern regions of China, and expand the scope of subjects to primary school students, college students and middle-aged and elderly people, so as to make the research results more representative. In addition, this study does not measure teenagers’ real leisure time and specific leisure behavior, but indirectly involves this problem in the form of questionnaire, which needs to be discussed in depth in the future.

## Conclusion

There are six typical correlation structures among leisure motivation, barriers, attitude, and satisfaction. Among them, leisure motivation has a significant positive correlation with leisure attitude and leisure satisfaction, and leisure attitude has a significant positive correlation with leisure satisfaction; Leisure barriers has a significant negative impact on leisure attitude, and the direct impact of leisure motivation on leisure attitude is significantly higher than that of leisure motivation on leisure satisfaction.

Leisure attitude is not only the intermediary between leisure motivation and leisure satisfaction, but also the intermediary between leisure barriers and leisure satisfaction. The variables of leisure motivation and leisure barriers can jointly explain 44% of the variation of leisure attitude, while the variables of leisure attitude, leisure motivation and leisure barriers can jointly explain 59% of the total variation of leisure satisfaction. After removing the negative effects of leisure barriers, it shows that, Leisure motivation is the determinant of leisure attitude and leisure satisfaction.

In terms of the impact of leisure motivation, barriers and attitude on leisure satisfaction, leisure motivation is the core variable, but leisure attitude plays a dual intermediary role. Therefore, it is possible that educating teenagers may establish a productive leisure attitude and improve leisure satisfaction.

## Data Availability Statement

The original contributions presented in the study are included in the article/supplementary material, further inquiries can be directed to the corresponding author/s.

## Ethics Statement

This study was reviewed and approved by the Ethics Review Committee of Chengdu Institute of Physical Education. However, this study does not involve human and animal experimentation, and written informed consent was not required.

## Author Contributions

YW was mainly responsible for the design of the manuscript and the preparation of the questionnaire and participates in the writing of the manuscript. JS, FF, and XW were mainly engaged in the distribution of the questionnaire and data processing and analysis. YP was mainly responsible for the coordination among the members of the research group, financial support, and revision of the manuscript. All authors contributed to the article and approved the submitted version.

## Conflict of Interest

The authors declare that the research was conducted in the absence of any commercial or financial relationships that could be construed as a potential conflict of interest.

## Publisher’s Note

All claims expressed in this article are solely those of the authors and do not necessarily represent those of their affiliated organizations, or those of the publisher, the editors and the reviewers. Any product that may be evaluated in this article, or claim that may be made by its manufacturer, is not guaranteed or endorsed by the publisher.
